# Supplementary material for: Seasonal Variation in the Rhizosphere and Non-Rhizosphere Microbial Community Structures and Functions of Camellia yuhsienensis Hu
Source: Microorganisms. 2020 Sep 10;8(9):1385. doi: 10.3390/microorganisms8091385 (PMC7564921; doi:10.3390/microorganisms8091385)
Supplement: Supplementary file 1 [file microorganisms-08-01385-s001.zip › Supplementary Material/Table S1 to S3.docx]

Table S1: Soil physicochemical property of each sample.

| Environmental factors | AR | BR | CR | DR | AN | BN | CN | DN |
| --- | --- | --- | --- | --- | --- | --- | --- | --- |
| AP (mg/kg) | 9.66±0.08a | 2.47±0.56b | 0.18±0.01c | 0.5±0.08c | 9.62±0.1a | 2.72±0.25b | 0.18±0.05c | 0.37±0.13c |
| TP (mg/kg) | 360.02±13.52ab | 397.68±41.5ab | 350.59±24.54b | 329.63±7.73b | 360.07±4.42ab | 422.96±10.8a | 369.02±1.71ab | 365.3±7.38ab |
| AK (mg/kg) | 90±3.4abc | 104.33±9.36a | 91.11±4.32abc | 70.67±9.96cd | 77.67±5.42bcd | 95±4.99ab | 78.56±6.29bcd | 64.11±6.04d |
| TK (mg/kg) | 287.67±9.3bc | 254±45.05c | 372.11±15.48a | 328±19.48abc | 303.33±19.35abc | 276.67±27.62bc | 373±14.07a | 338.67±11.7ab |
| AMN (mg/kg) | 41.55±1.08cd | 40.66±5.44d | 73.2±5.51ab | 58.63±5.53bc | 32.5±1.45d | 46.61±4.52c | 78.28±7.4a | 73.91±4.3ab |
| NN (mg/kg) | 9.64±1.06bc | 4.98±0.75c | 15.61±1b | 46±2.1a | 9.64±0.22bc | 6.52±0.97c | 17.22±0.07b | 41.11±6.1a |
| AHN (mg/kg) | 43.45±5.42c | 82.15±4.94b | 77.76±7.13b | 165.61±12.52a | 79.81±1.75b | 75.08±7.95b | 86.67±5.84b | 75.71±4.33b |
| TN (mg/kg) | 594.38±3.94a | 573.06±11.57a | 549.23±17.43a | 598.89±12.71a | 577.01±31.23a | 540.23±5.21a | 541.11±26.28a | 460.9±16.27b |
| TOC (g/kg) | 10.51±0.7ab | 12.49±0.9ab | 11.45±1ab | 13.01±1.08a | 9.89±0.32b | 12.39±0.44ab | 10.71±0.77ab | 10.24±0.34b |
| pH | 4.42±0.2a | 4.7±0.21a | 4.52±0.09a | 4.21±0.31a | 4.11±0.05a | 4.29±0.1a | 4.49±0.04a | 4.17±0.22a |
| SWC | 0.22±0.02ab | 0.22±0.03ab | 0.29±0.04a | 0.29±0.02a | 0.18±0.01b | 0.19±0.01b | 0.29±0.04a | 0.25±0.01ab |
| C/N | 17.66±1.07b | 21.8±1.48a | 20.76±1.2ab | 21.7±1.68a | 17.2±0.43b | 22.93±0.73a | 19.75±0.75ab | 22.24±0.37a |
| C/P | 29.53±3.12a | 33.29±6.02a | 33.85±5.63a | 39.3±2.46a | 27.44±0.64a | 29.34±1.18a | 29.01±2.04a | 28.12±1.43a |
| N/P | 1.66±0.07ab | 1.5±0.19abc | 1.6±0.17abc | 1.82±0.05a | 1.6±0.08abc | 1.28±0.04bc | 1.47±0.07abc | 1.27±0.07c |

AR, BR, CR and DR indicates rhizosphere soil sample in spring, summer, autumn and winter, respectively. AN, BN, CN and DN indicates non-rhizosphere soil sample in spring, summer, autumn and winter, respectively. AP, available phosphorous; TP, total phosphorous; AK, available potassium; TK, total potassium; AMN, ammonium nitrogen; NN, nitrate nitrogen; AHN, alkaline hydrolyzable nitrogen; TN, total nitrogen; TOC, total organic carbon; SWC, soil water content; Tem, soil monthly mean temperature; C/N, ratio of total carbon to total nitrogen; C/P, ratio of total carbon to total phosphorous; N/P, ration of total nitrogen to total phosphorous. Numbers before and behind “±” are mean value (N=3) and SE, respectively. Lowercases behind numbers indicate significant difference.

Table S2: Quantitative statistics of bacterial OTUs and Tags of each sample.

| Sample | Total Tags | Unique Tags | Taxon Tags | Unclassified Tags | Singleton Tags | OTUs |
| --- | --- | --- | --- | --- | --- | --- |
| AR-1 | 154058 | 117329 | 122520 | 0 | 31538 | 2167 |
| AR-2 | 124171 | 84174 | 108283 | 0 | 15888 | 1490 |
| AR-3 | 137444 | 116687 | 90468 | 0 | 46976 | 2212 |
| AN-1 | 135345 | 92972 | 118212 | 0 | 17133 | 1572 |
| AN-2 | 127700 | 80550 | 112873 | 0 | 14827 | 1443 |
| AN-3 | 140360 | 104654 | 118421 | 0 | 21939 | 1763 |
| BR-1 | 91851 | 52079 | 83926 | 0 | 7925 | 1096 |
| BR-2 | 91819 | 73114 | 67460 | 0 | 24359 | 1814 |
| BR-3 | 89072 | 64688 | 80100 | 0 | 8972 | 1374 |
| BN-1 | 89162 | 70101 | 69846 | 0 | 19316 | 1863 |
| BN-2 | 93801 | 59821 | 85807 | 0 | 7994 | 1351 |
| BN-3 | 97463 | 76726 | 81794 | 0 | 15669 | 1855 |
| CR-1 | 144616 | 117597 | 93328 | 0 | 51288 | 1957 |
| CR-2 | 124696 | 96657 | 86190 | 0 | 38506 | 1907 |
| CR-3 | 122305 | 88420 | 99693 | 0 | 22612 | 1717 |
| CN-1 | 90865 | 50563 | 83913 | 0 | 6952 | 995 |
| CN-2 | 101955 | 62486 | 91140 | 0 | 10815 | 1358 |
| CN-3 | 124471 | 86703 | 103318 | 0 | 21153 | 1734 |
| DR-1 | 177516 | 102521 | 168234 | 0 | 9282 | 1730 |
| DR-2 | 105162 | 53254 | 97134 | 0 | 8028 | 895 |
| DR-3 | 158934 | 99856 | 143311 | 0 | 15623 | 1795 |
| DN-1 | 162429 | 61971 | 155859 | 0 | 6570 | 777 |
| DN-2 | 153305 | 61147 | 144432 | 0 | 8873 | 671 |
| DN-3 | 175474 | 75434 | 167983 | 0 | 7491 | 926 |
| Average | 128037 | 87805 | 106953 | 0 | 21083 | 1720 |

AR, BR, CR and DR indicates rhizosphere soil sample in spring, summer, autumn and winter, respectively. AN, BN, CN and DN indicates non-rhizosphere soil sample in spring, summer, autumn and winter, respectively.

Table S3: Quantitative statistics of fungal OTUs and Tags of each sample.

| Sample | Total Tags | Unique Tags | Taxon Tags | Unclassified Tags | Singleton Tags | OTUs |
| --- | --- | --- | --- | --- | --- | --- |
| AR-1 | 188695 | 41439 | 183488 | 2379 | 2828 | 737 |
| AR-2 | 139884 | 25447 | 136472 | 2590 | 822 | 541 |
| AR-3 | 157680 | 26900 | 155046 | 194 | 2440 | 896 |
| AN-1 | 231015 | 37294 | 228474 | 385 | 2156 | 543 |
| AN-2 | 223034 | 38710 | 219082 | 658 | 3294 | 529 |
| AN-3 | 180863 | 42806 | 172039 | 60 | 8764 | 691 |
| BR-1 | 95722 | 18921 | 94227 | 248 | 1247 | 673 |
| BR-2 | 89041 | 24472 | 85557 | 308 | 3176 | 884 |
| BR-3 | 92039 | 19522 | 89413 | 1233 | 1393 | 688 |
| BN-1 | 91240 | 29262 | 85546 | 596 | 5098 | 901 |
| BN-2 | 93185 | 20500 | 91868 | 661 | 656 | 622 |
| BN-3 | 92456 | 21913 | 78635 | 12611 | 1210 | 719 |
| CR-1 | 127487 | 13910 | 126528 | 308 | 651 | 566 |
| CR-2 | 141974 | 34569 | 138298 | 519 | 3157 | 675 |
| CR-3 | 141747 | 12074 | 140479 | 96 | 1172 | 498 |
| CN-1 | 92346 | 7347 | 92190 | 4 | 152 | 234 |
| CN-2 | 97415 | 11969 | 97097 | 111 | 207 | 284 |
| CN-3 | 83522 | 13016 | 82303 | 1019 | 200 | 286 |
| DR-1 | 216660 | 40769 | 214610 | 436 | 1614 | 729 |
| DR-2 | 232095 | 40179 | 230364 | 629 | 1102 | 661 |
| DR-3 | 199106 | 32108 | 198497 | 228 | 381 | 678 |
| DN-1 | 210324 | 26083 | 209720 | 163 | 441 | 523 |
| DN-2 | 231188 | 28846 | 229942 | 129 | 1117 | 605 |
| DN-3 | 190120 | 23205 | 187994 | 1659 | 467 | 537 |
| Average | 154246 | 29819 | 151244 | 975 | 2025 | 677 |

AR, BR, CR and DR indicates rhizosphere soil sample in spring, summer, autumn and winter, respectively. AN, BN, CN and DN indicates non-rhizosphere soil sample in spring, summer, autumn and winter, respectively.
