# Supplementary figures and images for: Seasonal Variation in the Rhizosphere and Non-Rhizosphere Microbial Community Structures and Functions of Camellia yuhsienensis Hu
Source: Microorganisms. 2020 Sep 10;8(9):1385. doi: 10.3390/microorganisms8091385 (PMC7564921; doi:10.3390/microorganisms8091385)

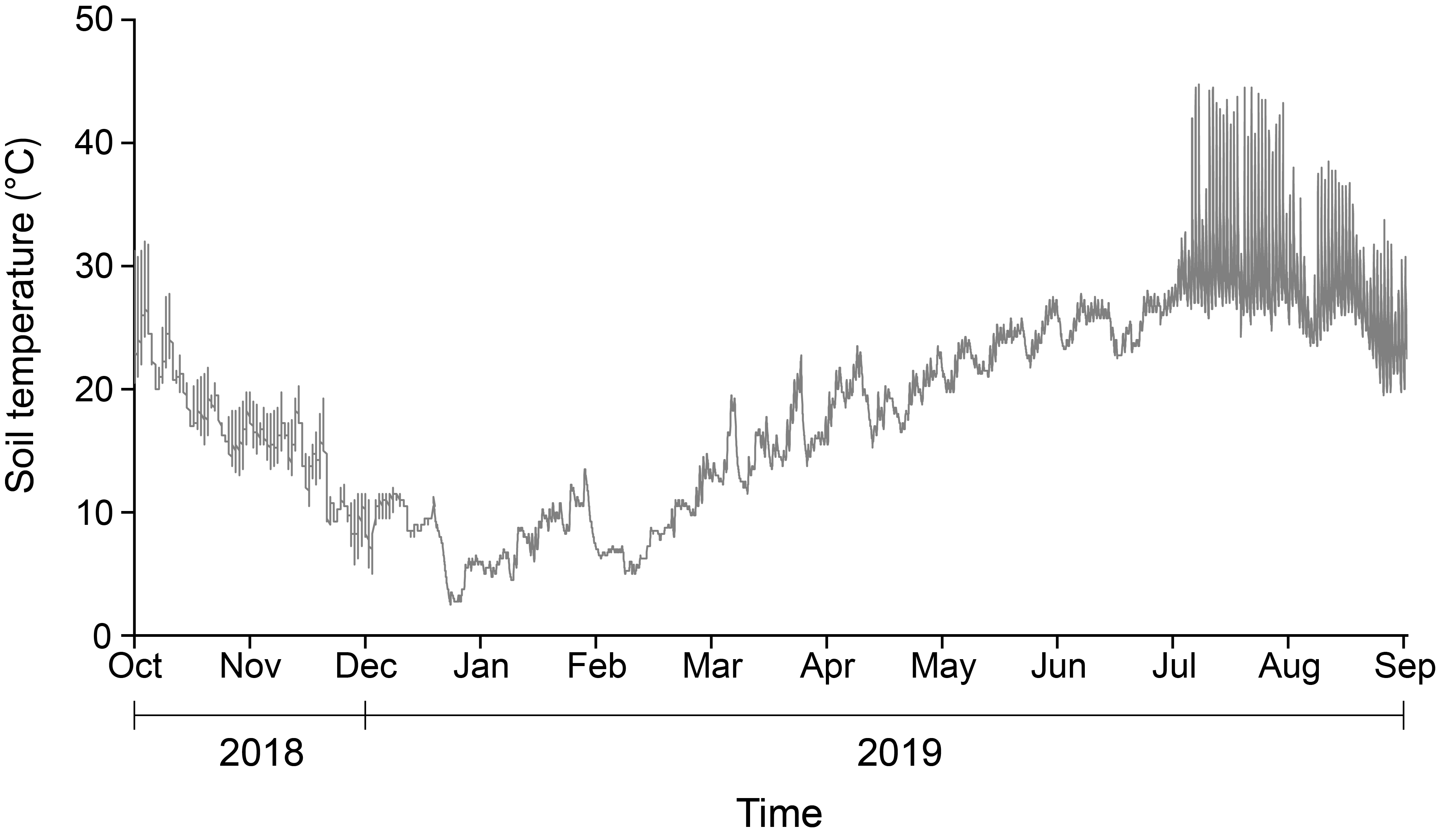

Supplement: Supplementary file 1 [file microorganisms-08-01385-s001.zip › Supplementary Material/Figure S1.jpg]

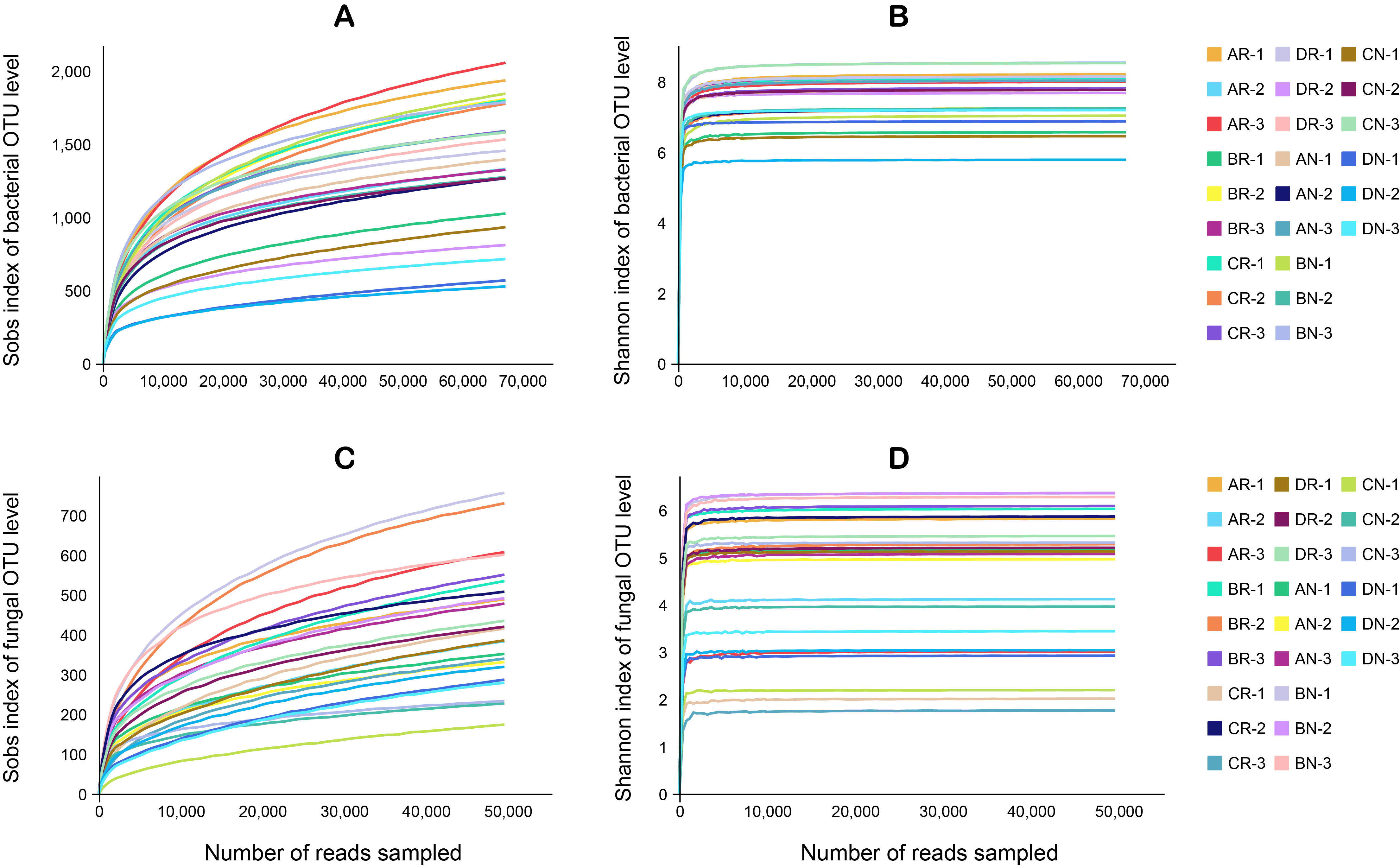

Supplement: Supplementary file 1 [file microorganisms-08-01385-s001.zip › Supplementary Material/Figure S2.jpg]

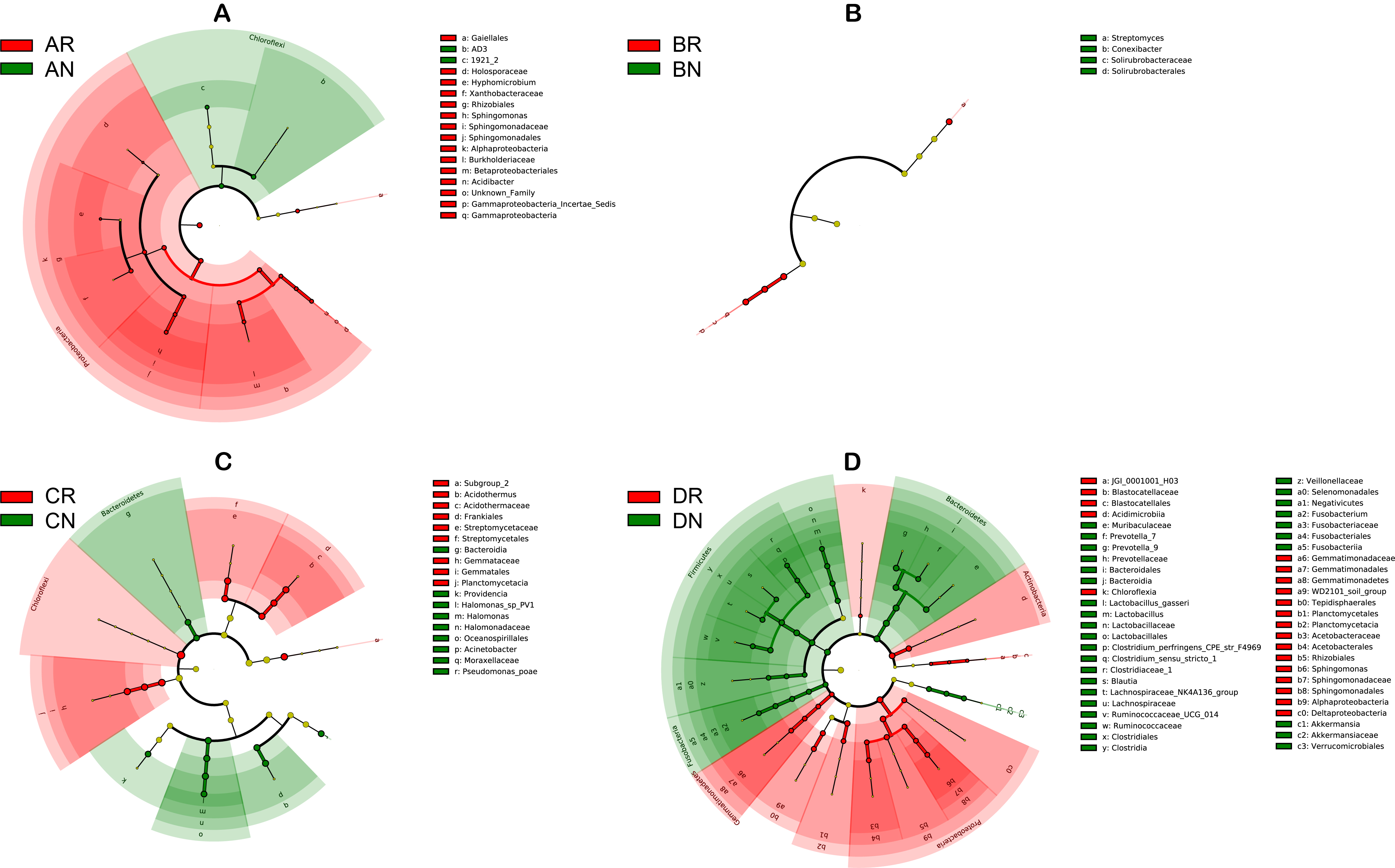

Supplement: Supplementary file 1 [file microorganisms-08-01385-s001.zip › Supplementary Material/Figure S3.jpg]

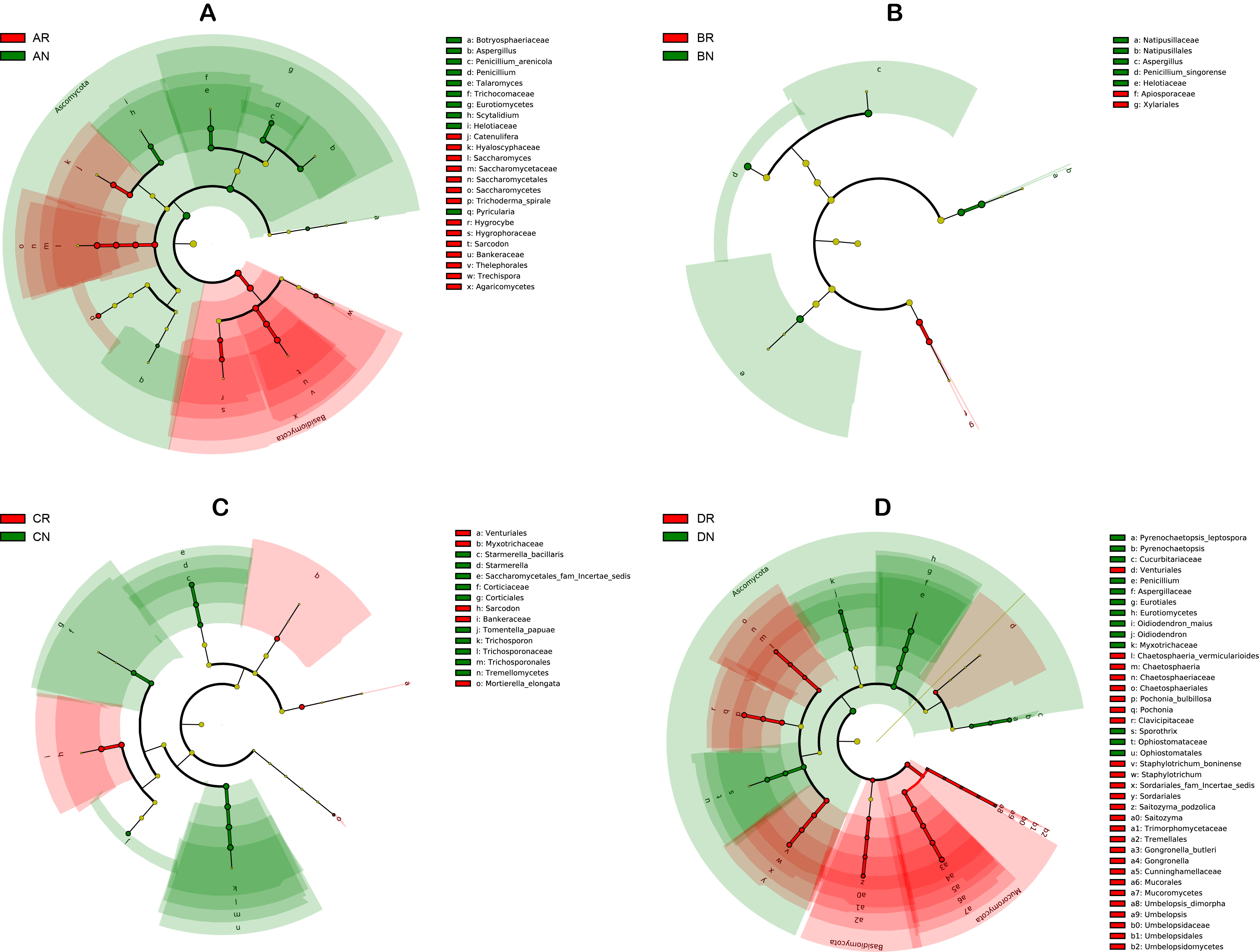

Supplement: Supplementary file 1 [file microorganisms-08-01385-s001.zip › Supplementary Material/Figure S4.jpg]

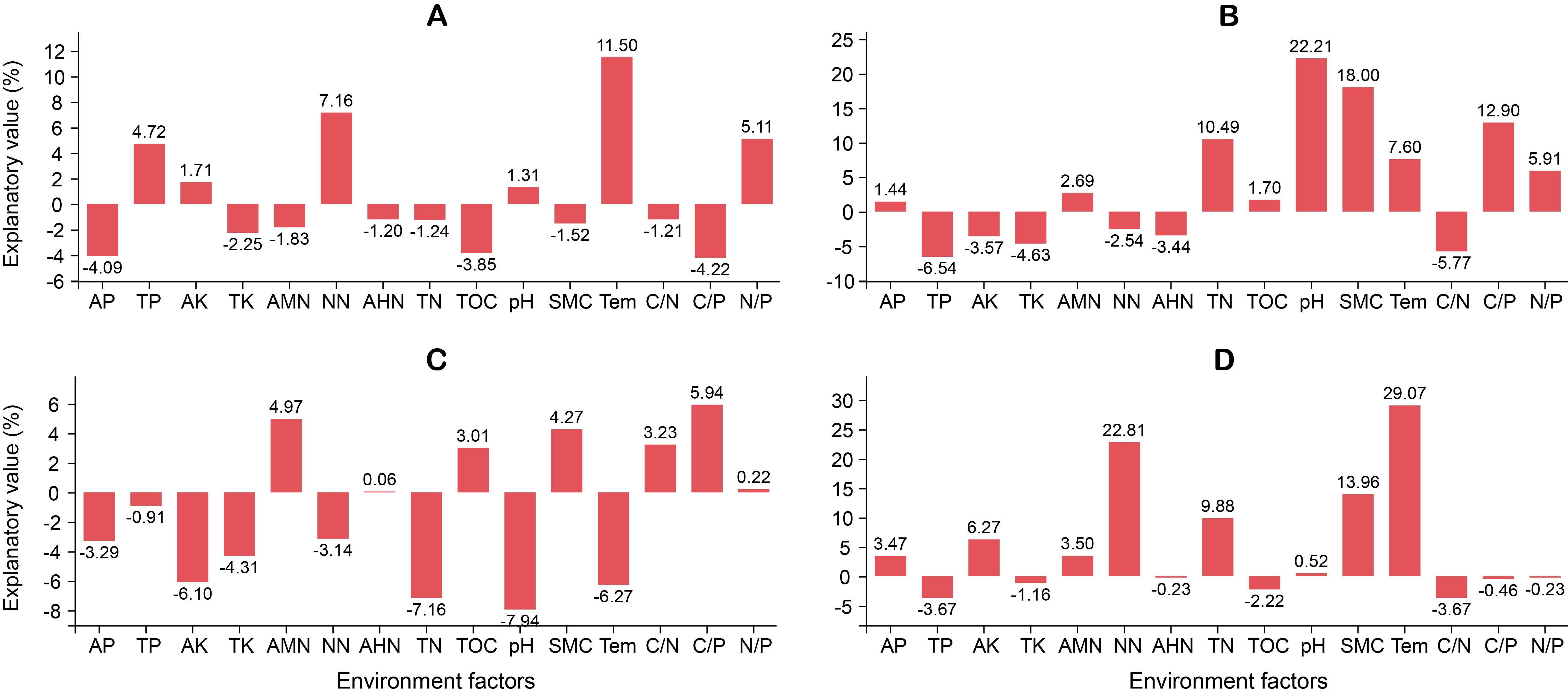

Supplement: Supplementary file 1 [file microorganisms-08-01385-s001.zip › Supplementary Material/Figure S5.jpg]

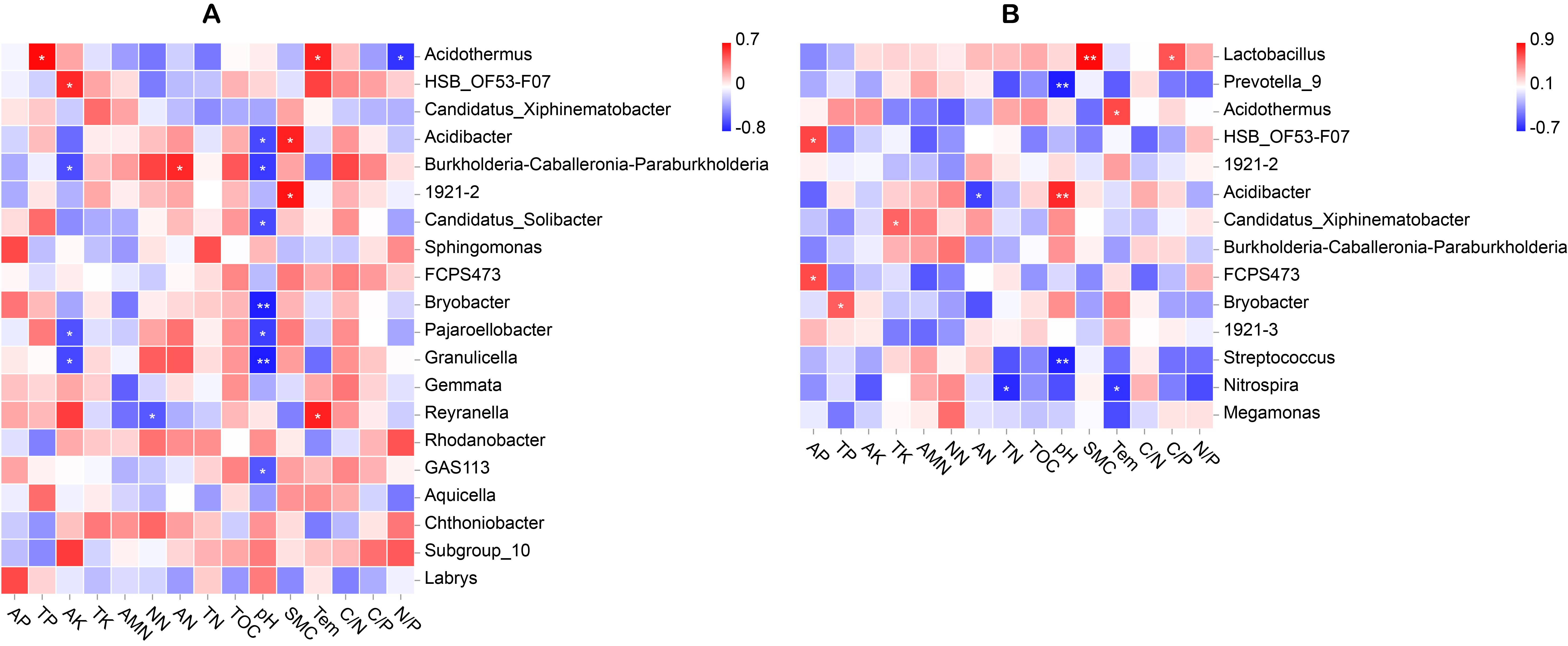

Supplement: Supplementary file 1 [file microorganisms-08-01385-s001.zip › Supplementary Material/Figure S6.jpg]

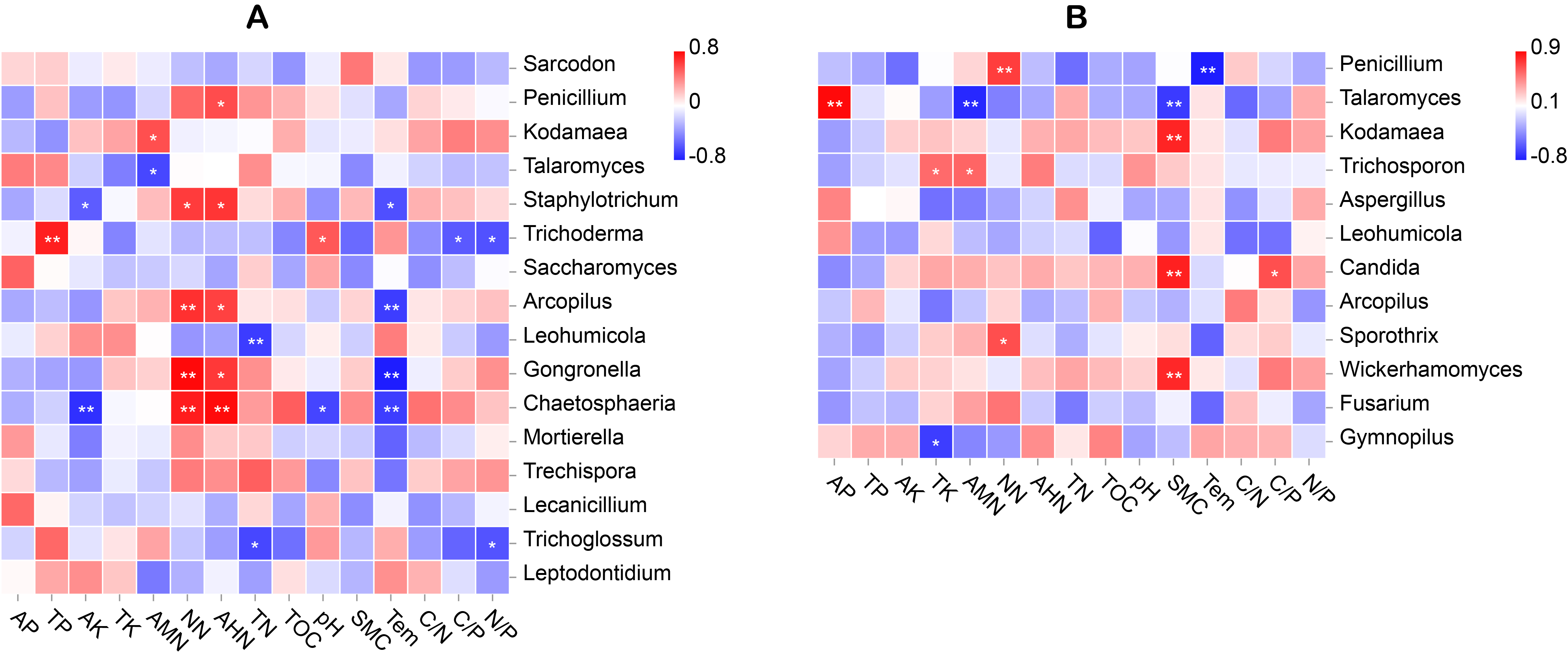

Supplement: Supplementary file 1 [file microorganisms-08-01385-s001.zip › Supplementary Material/Figure S7.jpg]
